# Perceptions, Benefits, and Use of Complementary and Integrative Therapies to Treat Menopausal Symptoms: A Pilot Study

**DOI:** 10.1089/whr.2022.0105

**Published:** 2023-03-27

**Authors:** Marnie L. Vanden Noven, Mia Larson, Emma Lee, Cavan Reilly, Mary Fran Tracy, Manda L. Keller-Ross

**Affiliations:** ^1^ Department of Exercise Science, College of Health Sciences and Nursing, Belmont University, Nashville, Tennessee, USA.; ^2^ Medical Specialists of Kentuckiana, Owensboro, Kentucky, USA.; ^3^ Division of Physical Therapy, Medical School, University of Minnesota, Minneapolis, Minnesota, USA.; ^4^ Division of Biostatistics, School of Public Health, University of Minnesota, Minneapolis, Minnesota, USA.; ^5^ Adult and Gerontological Health Cooperative, School of Nursing, University of Minnesota, Minneapolis, Minnesota, USA.

**Keywords:** exercise, vasomotor, complementary alternative medicine, sleep disturbances, perimenopausal, integrative health

## Abstract

**Background:**

Menopause symptoms can be debilitating, and the use of menopausal hormone therapy (MHT) has declined significantly since the Women's Health Initiative.

**Materials and Methods:**

We surveyed 508 peri- and postmenopausal females to determine (1) the use of complementary and integrative therapies (CIT), MHT; and pharmacotherapies; (2) the perceptions, perceived benefits/risks of CIT, MHT; and pharmacotherapy use; and (3) factors associated with CIT and MHT use for menopause symptom treatment.

**Results:**

The majority of respondents used CIT to treat menopause symptoms based on physician recommendation and research studies. Treatments that were perceived as most beneficial included exercise, mind–body therapies, diet, and spiritual practices, with exercise and mind–body therapies chosen to treat the most common symptoms of sleep disturbances, depressive mood, and anxiety. Higher education level was the main predictive variable for choosing exercise (odds ratio [OR] = 1.27, *p* = 0.02) and mind–body therapies (OR = 1.57, *p* = 0.02) to treat menopausal symptoms. Perceptions, beliefs, and use of different CIT by primarily white, affluent, and educated peri- and postmenopausal females to treat menopause symptoms, including sleep disturbances, depression, and anxiety, are driven by conversations with physicians and evidence-based research.

**Conclusion:**

These findings reinforce the necessity for both additional research in more diverse populations, as well as comprehensive, individualized personalized care from an interdisciplinary team that considers the best options available for all female patients.

## Introduction

Menopausal symptoms, such as insomnia, vasomotor symptoms (VMS, hot flashes, night sweats, sweating),^1^ mood and sleep disorders, and memory difficulties,^2^ can be debilitating and significantly influence quality of life.^3^ Over 70% of females will experience at least some of these symptoms.^4^ Menopausal hormone therapy (MHT) was a standard and effective treatment for menopausal symptoms until the 2002 Women's Health Initiative reported a greater risk-benefit ratio for breast cancer, stroke, and other disorders.^5^ Despite subsequent studies demonstrating MHT safety for perimenopausal females up to 10 years following menopause onset, symptom management using MHT remains low^6–8^ and 47% of middle aged females still report a preference for not taking MHT.^1^

Studies published since 2002 estimate increased complementary and integrative therapies (CIT) use,^9,10^ including findings that 89.7% of the 79.3% females discontinuing MHT had used CIT for menopausal symptoms,^11^ and others estimating CIT use between 31% and 82%.^12,13^ Despite evidence of increased CIT use, prevalence, perceptions, and beliefs regarding CIT effectiveness in menopausal females are poorly understood. The purpose of this study was to determine (1) CIT, MHT, and other pharmacotherapy use for menopausal symptom treatment; (2) CIT, MHT, and other pharmacotherapy perceived risks and benefits for menopausal symptom treatment; and (3) factors associated with increased or decreased CIT and MHT use for menopausal symptoms.

## Methods

A convenience sample of peri- and post-menopausal females >35 years old completed a pilot, cross-sectional design survey in collaboration with the University of Minnesota Driven to Discover Program at the 2019 Minnesota State Fair. Menopause is defined by 12 consecutive months without menses. Younger females (>35 years old) may experience menopausal symptoms during perimenopause (onset of menopause-related symptoms and menstrual cycle changes) or premature menopause (at or before the age of 40) and were included in this study.^14^ The University of Minnesota Institutional Review Board approved the study protocol (IRB No. 00006540), and the study was conducted in accordance with the Declaration of Helsinki.

### Instrument

A previous survey assessing CIT perceptions and beliefs in nurses^15,16^ was modified (with permission) to determine CIT use in females experiencing menopause-related symptoms. Therapies utilized (*n* = 28) followed complementary therapies reported by the National Center for Complementary and Integrative Health.^17^

### Demographics, health, and social history

Demographics included: race, education, employment, marital status, annual income, and religion. Participants reported their primary health care provider and frequency of visits. Female health, menopause, and cancer history, including gynecological and breast cancer, chemotherapy, MHT use, and pregnancy histories, were collected. Menopause history included age, menopause onset age, history, and reason for hysterectomy and/or oophorectomy. Average stress level (Likert scale 1–10, 1 = *little to none* to 10 = *most possible stress*); history and frequency of nicotine, caffeine, and alcohol use; history of MHT use/nonuse, rationale, forms of MHT used (pills, creams, sprays), rationale for using/not using MHT; and any nonhormone medications used to treat menopause symptoms were also collected (Table 1).

**Table 1.** Menopausal Hormone Therapy and Nonmenopausal Hormone Therapy Options, and Reasons for Using and Not Using Menopausal Hormone Therapy to Treat Menopause Symptoms

MHT options	Reason for MHT use	Non-MHT options	Reason for not using MHT
Estrogen pills	My doctor prescribed it	Clonidine (Catapres)	Never considered
Estrogen and progestin pills	Heart disease prevention	Citalopram (Celexa)	Doctor recommendation
Estrogen patch	Menopause symptom treatment	Escitalopram (Lexapro)	I do not need it
Estrogen and progestin patch	Prevent weight gain or lose weight	Fluoxetine (Prozac)	I wouldn't benefit from it
Estrogen cream or spray	Improve skin, hair, nails	Paroxetine (Paxil, Pexeva)	Increased cancer risk concern
Phytoestrogen	Improve bone health, prevent bone loss	Sertraline (Zoloft)	Increased cardiovascular disease risk concern
Oral contraceptives	Other reason	Vilazodone (Viibryd)	Stopped due to side effects
		Gabapentin (Neurontin)	Doctor has never suggested
		Other	Other reason

MHT, menopausal hormone therapy.

### Physical activity

Participants reported intensity and hours spent exercising (*i.e.*, 6+, 4½–6, 2½–4, ½–2, <½, or 0 hours) per week. Strenuous exercise caused a rapid heartbeat from activities like aerobics, jogging, or swimming laps. Moderate exercise was not exhausting and included examples like walking quickly or easy biking. Mild exercise required little effort and included walking slowly and golf.

### Menopause quality of life

Menopause quality of life was determined using the menopause-specific quality of life questionnaire (MENQOL),^18^ which identifies four menopausal symptom domains experienced over the last 6 months: VMS, psychosocial, physical, and sexual. Items were rated as present/not present, and if present, how bothersome on a zero (not bothersome) to six (extremely bothersome) Likert scale. Absent symptoms are scored as a “1,” and present symptoms are scored as a “2” plus the bothersome rating, with the total score of each domain ranging from one to eight. Domain means are computed individually for the final score. Domain contributions vary, so there is no cumulative score.^18,19^

### CIT use evidence

Participants indicated importance of evidence for selecting CIT to treat menopause symptoms using a 4-point Likert scale (unimportant-important). Options included: research studies by physicians or large academic institutions; doctor's recommendation; news media; information on a blog, popular journal, or similar website; medical website such as WebMD or the Mayo Clinic; alternative medicine practitioner's advice; successful use on themselves; or advice from a trusted friend or family member.

### Perceived benefit or harm

Participants indicated CIT benefit/harm beliefs for menopause symptom treatment using a Likert scale of 1 (“Harmful”) to 5 (“Beneficial”). Therapies included the following: MHT, non-MHT, other medications, diet, mental health, exercise, mind–body therapy, spiritual practices, energy therapies, or integrative therapies (Table 2).

**Table 2.** Options for Complementary and Integrative Therapies Women Could Indicate They Had Ever Used to Treat Their Menopausal Symptoms

MHT	Integrative therapies	Non-MHT	Diet, vitamin, herbal supplements	Mental health therapies
Pills containing estrogen such as Cenestin, Estrace, Estratab, Femtrace, Ogen, Permarin, estrogen-bazedoxifene, or Duavee.	Homeopathic Medicine	Clonidine (Catapres)	Added foods to diet	Behavioral medicine
	Native American Medicine, Shamanism or other tribal medicine	Citalopram (Celexa)	Removed foods from diet	Biofeedback
		Escitalopram (Lexapro)	Intentionally lost weight	Relaxation techniques
	Naturopathic Medicine	Fluoxetine (Prozac)	Increased soy content of diet	Counseling/psychotherapy
Pills containing estrogen and progestin such as Activella, FemHrt, and Prempro	Ayurveda (Traditional Indian Medicine)	Paroxetine (Paxil, Pexeva)	Vitamins	Mindfulness program
		Sertraline (Zoloft)	Herbal supplements	Group therapy
	Traditional Chinese Medicine	Vilazodone (Viibryd)	Black Cohosh	Hypnotherapy
A patch containing estrogen such as Alora, Climara, Estraderm, or Vivelle-Dot.	Electromagnetic/Magnet Application	Gabapentin (Neurontin)	Red Clover	
	Acupuncture	Other	Ginseng	
	Chiropractic Therapy		Flavonoids or isoflavones	
A patch containing both estrogen and progestin such as Climara Pro Combipatch.	Environmental medicine		Menopause support supplement (Amberen, Estroven, Staying Cool, Dr. Tobias, or similar)	
	Art Therapy			
	Music Therapy			
A cream or spray containing estrogen such as Estroge, Divigel, Estrasorb, or Evamist	Hypnotherapy		Evening Primrose	
	Other		Phytoestrogen (a plant-based estrogen supplement)	
		
Phytoestrogen (a plant based estrogen supplement)			Other	
			
Oral contraceptives				

Mind–body therapies		Exercise	Energy therapies	Spiritual practices
Massage	Rolfing	Walking	Tai Chi	Prayer and Spiritual Direction
Physical therapy	Structural integration	Running/Jogging	Chi (Qi) Gong	Meditation
Breathing exercises	Kinetic body therapy	Swimming	Reiki	Mindfulness-based stress reduction or other mindfulness program
Yoga	Alexander technique	Water aerobics	Healing Touch	
Meditation	Feldenkrais method	Biking	Micro current	
Mindfulness	Hanna Somatic technique	Group fitness classes	Electromagnetic application	Attending spiritually oriented group gatherings such as group meditation, church, temple, or a mosque
Hypnotherapy	Acupuncture	Strength training exercise	Acupuncture	
Guided imagery	Acupressure	Yoga	Other	
Myofascial release	Other body movement therapy	Pilates		Other
Trigger point therapy		Team sports (tennis, soccer, etc…)		
		Other		

### Personal use, perceived effectiveness

Complementary and integrative therapies use history included MHT, non-MHT, other medications, diet, mental health, exercise, mind–body therapy, spiritual practices, energy therapies, or integrative therapies. Options included the following: Yes; No; No, but have considered using or have interest in using; or I have used this therapy but not for menopause symptoms. Participants indicated CIT effectiveness (“effective” was not defined for participants), specific CIT type used, and symptom(s) the CIT effectively treated.

### Analysis

Descriptive statistics described demographic data, health and social history, physical activity, CIT use evidence, personal CIT use, perceived benefits/harm and effectiveness, and menopause history. Percentages of use and perceived effectiveness were calculated for treatment categories. Means and standard deviations were used to summarize continuous variables. MENQOL scores were calculated and reported as described above. Logistic regression was used to estimate a participant's treatment choice as related to MENQOL scores.

One-way ANOVA analysis was used to compare interactions between demographic data (age, education, employment, marital status, and annual income) and therapy effectiveness beliefs; exercise choice, income levels, and age and therapy effectiveness beliefs; and MENQOL scores and age. A binary logistic regression analysis was performed to determine if demographic variables were predictors for commonly chosen treatment methods. The resulting odds ratio (OR) and estimated conditional probabilities illustrated which demographic variables influenced CIT use for menopausal treatment. *p*-values <0.05 were statistically significant.

## Results

### Demographics, health history, and physical activity

Of the 508 surveys initiated, 474 (93.3%) were analyzed and 39 were excluded (due to being incomplete) (Table 3). Participants saw their physician for primary health care needs once a year (56.8%). Health concerns included high blood pressure (27.6%), anxiety (22.3%), heavy menstrual bleeding (21.9%), depression (21.5%), and high cholesterol (18.7%). Mean stress levels were 4.9 ± 2.0 (on a 0 to 10 scale). Current nicotine/tobacco use (4.7%) included cigarettes (77.3%), e-cigarettes (9.1%), tobacco (4.5%), and other products (9.1%); 17.7% reported past cigarette (97.4%) or other tobacco product (2.6%) use. Most consumed caffeinated beverages were coffee (67.8%) or caffeinated soda (17.8%), daily. Participants (59.2%) consumed one to two alcoholic beverages per occasion (89.6%), two to four times per month (37.8%) or two to three times per week (34.2%), and most (79.9%) had never had more than six drinks on one occasion. Participants completed an average 2 hours or less of mild (60.5%), moderate (67.3%), or strenuous activity (46%) per week.

**Table 3.** Participant Characteristics and Demographics

Demographic	Results (mean ± SD or %)		
Age (years)	55 ± 7		
Weight (lbs.)	170 ± 40		
Height (in.)	64.8 ± 3.7		
Body mass index (kg/m^2^)	28.7 ± 7.3		

Demographic	Options	*N*	%
U.S.-born		439	92.4
MN resident		392	82.5
WI resident		16	3.4
Race	White or Caucasian	448	94.7
	Hispanic, Latino, or Spanish	3	0.6
	Black or African	4	0.8
	Asian or Asian Indian	8	1.7
	Other/multiracial	10	2.1
Education	High school diploma	50	10.5
	Technical college/Associate degree	102	21.5
	Bachelor's degree	168	35.4
	Graduate degree	154	32.5
Employment	Full-time	293	61.8
	Part-time	85	17.9
	Not employed	96	20.3
Marital status	Married	358	76.5
	Single	45	9.6
	Divorced	55	11.8
	Widowed	10	2.1
Income	Less than $30,000	15	3.2
	$30,000–64,999	75	16
	$65,000–100,000	155	33.1
	Over $100,000	222	47.4
Religion	Christian	382	80.9
	Jewish	4	0.8
	Hindu	1	0.2
	Buddhism	2	0.4
	Not religious/Atheist	23	4.9
	Agnostic/Spiritual but not religious	41	8.7
	Other	19	4

### Menopause and MENQOL

Most participants (65.7%, *n* = 309) had gone through menopause, while some (15.1%, *n* = 71) were currently perimenopausal. The last normal menstrual cycle occurred at 48 ± 7 years. Average hysterectomy age was 45 ± 8 years (16.1%, *n* = 76), and oophorectomy was 47 ± 8 years (9.5%, *n* = 45). Hysterectomy and oophorectomy were reported by 6.3% (*n* = 30) of participants due to uterine fibroids (23%, *n* = 17), other reason not specified (17.6%, *n* = 13), endometriosis (14.9%, *n* = 11), or a total hysterectomy (10.8%, *n* = 8). Average MENQOL scores were: VMS = 3.37, psychosocial = 3.76, physical = 3.86, and sexual = 2.44.

When describing what they did when choosing to treat menopause symptoms, participants consulted a health care practitioner (40.5%, *n* = 192), added exercise or dietary changes (32.9%, *n* = 156), and sought support from other females who have or are going through menopause (15.6%, *n* = 74). Some participants used over the counter supplements or herbal treatments (13.5%), while others had done nothing (30.4%) or had not experienced menopause symptoms (11%).

Participants with VMS were significantly more likely to choose more forms of treatment than their counterparts without VMS, including exercise (OR = 1.50, *p* = 0.0001), MHT (OR = 1.37, *p* = 0.003), diet (OR = 1.47, *p* = 0.0001), mind–body therapies (OR = 1.31, *p* = 0.03), movement therapies (OR = 1.21, *p* = 0.049), energy therapies (OR = 1.47, *p* = 0.02), and integrative therapies (OR = 1.28, *p* = 0.045). Participants experiencing sexual symptoms were significantly more likely to choose MHT (OR = 1.28, *p* = 0.004) and movement therapies (OR = 1.19, *p* = 0.02) than their counterparts.

### Personal use and perceived effectiveness

The majority of females (59%) reported using an average of 1.8 ± 2.8 (range 0–9) different types of CIT. Participants most commonly chose exercise (*n* = 219), mind–body therapies (*n* = 135), diet (*n* = 98), and spiritual practices (*n* = 91) for treatment. Most considered CIT at least moderately effective (59.9%), very effective (27.9%), or not effective (12.2%). When asked how effective specific CIT were in treating menopause symptoms, 93.3% of participants using energy therapies felt they were moderately to very effective, followed by mind–body therapies (91.9%), exercise (91.3%), spiritual practices (90.1%), and diet (73.5%).

**FIG. 1.**
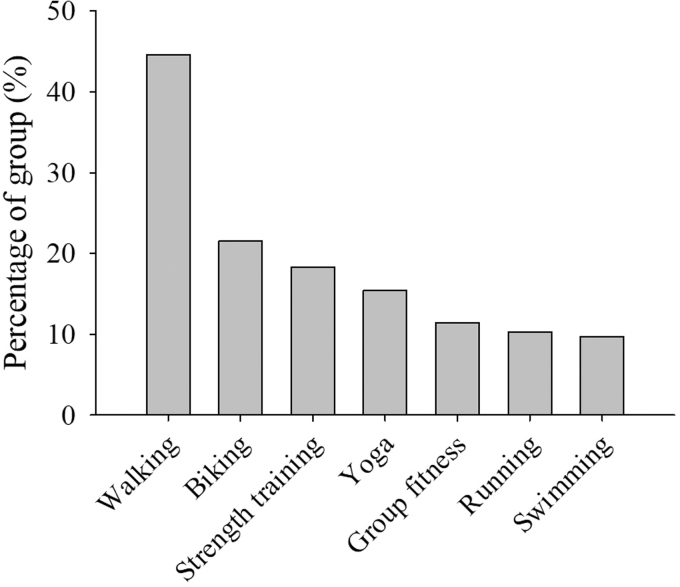
Exercises chosen for menopause symptom treatment reported by participants as percent of total population (*n* = 374).

**FIG. 2.**
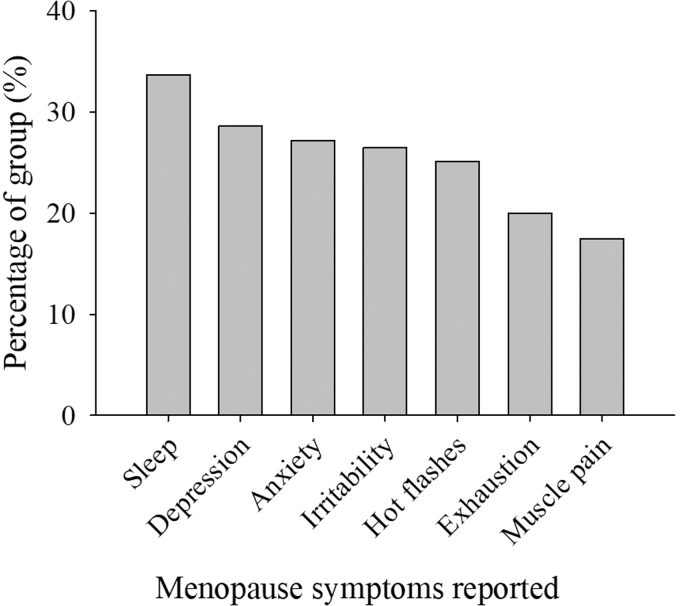
Menopause symptoms reported by participants as percent of total population (*n* = 374).

**FIG. 3.**
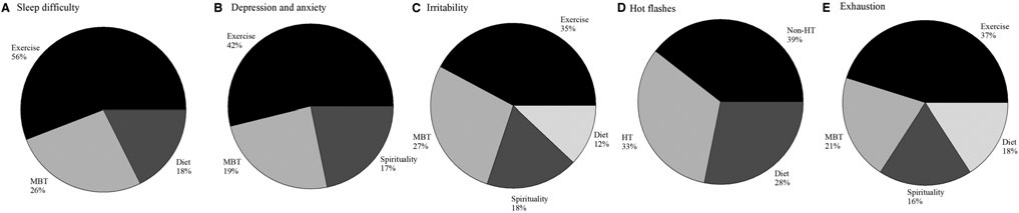
Most common menopause symptoms treated by participants, **(A)** sleep problems, **(B)** depression and anxiety, **(C)** irritability, **(D)** hot flashes, and **(E)** exhaustion, and the frequency of the most common therapies they chose to treat, including exercise, mind–body therapies, diet, MHT, and spiritual practices. MHT, menopause hormone therapy; MBT, mind-body therapy.

**FIG. 4.**
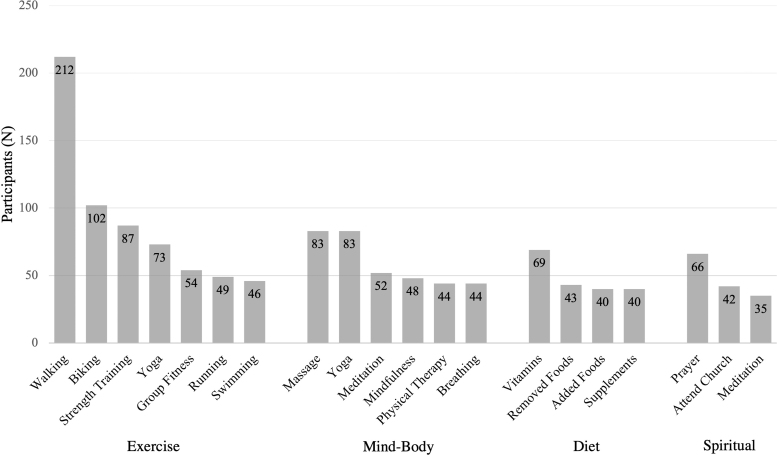
Most common practices chosen by participants to treat menopause symptoms for exercise, mind–body therapies, diet, and spiritual practices.

Participants most often chose walking (*n* = 212), biking (*n* = 102), strength training (*n* = 87), and yoga (*n* = 73) exercises for menopause symptom treatment (Fig. 1). Participants chose to treat sleep problems (33.7%), depressive mood (28.6%), and anxiety (27.2%) the most (Fig. 2), using exercise, mind–body therapies, diet, and spiritual practices. They also treated irritability (26.5%), hot flashes (25.1%), and exhaustion (20%) using exercise, mind–body therapies, diet, and spiritual practices (Fig. 3). The most common exercise, mind–body, diet, MHT, and spiritual practices are illustrated in Figure 4.

Education was the strongest predictive variable associated with the treatment individuals chose for menopausal symptoms. Indeed, participants achieving higher education levels were more likely to choose exercise (OR = 1.27, *p* = 0.02) or mind–body therapies (OR = 1.57, *p* = 0.02). Females with a college degree were significantly more likely to choose exercise to treat menopause symptoms than their peers (OR = 2.38, *p* = 0.009), and those with graduate degrees were significantly more likely to choose mind–body therapies to treat menopause symptoms (OR = 2.36, *p* = 0.01) than individuals without graduate degrees.

### Types of evidence required

Participants reported that doctors' recommendations (74.5%, *n* = 353) and “successful use myself” (63.5%, *n* = 301) were important/somewhat important when choosing menopause symptom treatment. Research studies by physicians or large academic institutions supporting therapy effectiveness (63.3%, *n* = 300) and research evidence by physicians or large academic institutions suggesting that the therapy may be effective (but has not yet been proven) (49.2%, *n* = 233) were also important for decision-making. Unimportant/somewhat unimportant factors when making treatment decisions included news media reports that the treatment works (58.2%, *n* = 276) and information on a blog, popular journal, or similar website suggesting the effectiveness of the therapy (55.1%, *n* = 261). Information on a medical website such as WebMD, Harvard Health, or Mayo Clinic suggesting benefit of the therapy (52.1%, *n* = 247) was also considered unimportant/somewhat unimportant.

### Perceived benefits/harm of therapies

Most participants (69%, *n* = 320) thought that exercise (perceived benefit: 4.4 ± 1.2) and mind–body therapies (62.1%, *n* = 288) were beneficial (4.2 ± 1.3) and considered harmful therapies to be MHT (17.3%, *n* = 81, 2.5 ± 1.7) and other nonhormone pharmacotherapies (6.9%, *n* = 32, 2.1 ± 1.7). Oral contraceptives (8.4%, *n* = 40), estrogen pills (8%, *n* = 38), and estrogen progestin patch (4.4%, *n* = 21) were the MHTs that were most used. Participants used MHT because their doctor prescribed it (13.3%, *n* = 63) to treat menopause symptoms (9.7%, *n* = 46) or other issues (birth control, heavy menstrual cycles; 3.6%, *n* = 17). When asked why they were not taking MHT, participants never considered it (26.3%, *n* = 125), did not need it (17.1%, *n* = 81), were concerned about an increased cancer risk (12.4%, *n* = 59), or their doctor never suggested using MHT (12.2%, *n* = 58).

The effect of education on therapy effectiveness beliefs revealed that there was a significant difference in therapy effectiveness beliefs in non-MHT across education levels for exercise [*F*(3, 46) = 10.07, *p* < 0.001]; mind–body therapies [*F*(3, 460) = 6.46, *p* < 0.001]; mental health therapies [*F*(3, 464) = 3.23, *p* = 0.02]; and spiritual practices [*F*(3, 457) = 4.43, *p* = 0.004]. *Post hoc* analysis revealed that individuals with a technical degree or higher believed that exercise, mind–body, mental health therapies, and spiritual practices were more effective in treating menopause symptoms than females with a high school diploma. Income and age did not influence beliefs of therapy effectiveness (*p* > 0.05).

## Discussion

Most participants used at least one CIT to treat menopause symptoms and relied on physicians' recommendations and research evidence to assist with decision-making. Exercise and mind–body therapies were most chosen to treat menopause symptoms, perceived as beneficial, and used most often to treat sleep disturbance, the most common menopause symptom. Furthermore, education most influenced CIT selection for menopause symptom treatment.

### Exercise and mind–body therapies

This cohort found exercise and mind–body therapies useful in treating sleep problems, depression, and anxiety. Exercise benefits have been clearly demonstrated; however, evidence-based effectiveness of mind–body therapies is equivocal. Exercise improves cardiorespiratory capacity, weight preservation/loss (with diet), bone mineral density, and muscle strength in postmenopausal females.^20^ Furthermore, walking programs improve cardiovascular markers, lipid and carbohydrate metabolism disorders (thereby decreasing hypertension),^20,21^ physical and mental health, and sexual quality of life.^22^

Exercise can be a safe, useful intervention strategy to alleviate menopause symptoms.^23^ Mindfulness, massage, and yoga may be beneficial for reducing insomnia, VMS, and depression^24–27^; however, there is insufficient evidence for long-term VMS relief using mind–body therapies in healthy menopausal females.^28^ Furthermore, psychological and behavioral therapies only moderately reduce VMS.^29^ Thus, exercise programs can provide significant symptom relief and health benefits but mind–body therapy use should be explored with a well-informed health care professional.

### Sleep disturbances and menopause

Sleep difficulty prevalence can range from 34% to 60% in peri- and post-menopausal females (33.7% in this cohort) and increases with age.^30–32^ Although complex, poor sleep etiology is closely related to menopause symptoms, such as hot flushes.^32,33^ Females experiencing sleep difficulties are at greater risk of depression^34^ and cardiovascular risk markers than females without sleep difficulties.^35,36^ Furthermore, VMS directly correlate with insomnia and depression and are primary predictors of sleep problems in menopausal females,^37^ often resulting in sleep deficiencies, irritability, and mood changes.^38^

Fortunately, evidence-based treatment for menopause-related sleep disturbances is well supported. Pharmacological management of postmenopausal insomnia may include MHT, selective serotonin reuptake inhibitors (SSRIs), and dual serotonin and norepinephrine reuptake inhibitors (SNRIs)^39^; however, cognitive behavioral therapy (CBT) for insomnia (CBT-I) may be the gold standard for insomnia treatment.^40^ Moderate-to-low intensity exercise can also improve sleep quality and quality-of-life scores and reduce nighttime hot flushes in postmenopausal females.^41,42^ Thus, interrelated sleep and menopause symptom management may warrant pharmacological, CBT-I, and exercise treatment strategies.^40^

### Depression, anxiety, and menopause

Upwards of 70% of perimenopausal females will experience depressive symptoms compared with premenopausal females, which can be disruptive and decrease quality of life.^43,44^ Increased risk of major depressive disorder (MDD) development or recurrence may be related to prior MDD history, prior anxiety diagnosis, being peri- and post-menopausal, hormonal status changes,^45^ and history of VMS.^46^

Consensus recommendations for perimenopausal depression treatment include SSRIs and SNRIs, which may also improve VMS^47^; a combination of CBT and antidepressant pharmacotherapy can effectively decrease depression symptoms and improve recovery rates and treatment compliance.^48^ Ultimately, encouraging exercise while concurrently addressing depression, sleep disturbances, and VMS in both peri- and postmenopausal females may be the most effective treatment strategy.^47^ Limited support for hypericum perforatum (St. John's wort) treatment of mild/moderate depression exists; however, it is less effective with severe depression and comes with multiple safety concerns.^49^

Estradiol treatment may be beneficial depression treatment for menopausal females.^44^ The “window of vulnerability,” during which females experience increased sensitivity to hormonal changes that could contribute to depressive symptoms and MDD development,^50,51^ coincides with the timing hypothesis “window of opportunity,”^52^ during which MHT lowers coronary heart disease and atherosclerosis incidences, decreases mortality, and improves quality of life in perimenopausal females. Furthermore, MHT antidepressant effects in perimenopausal females can persist despite VMS reemergence^53^ and may provide some value for depression and VMS symptom prevention or for patients unwilling or unable to utilize antidepressants; however, MHT is not recommended for late- postmenopausal females^47,54,55^ and is not approved to treat depression in the United States or Europe.^49^

Despite anxiety symptom reports as high as 52% in 40–55 year old females,^56^ many challenges make diagnosis and treatment challenging,^57–61^ such as clustering anxiety symptoms with other psychological symptoms;^56,62^ unclear relationships between anxiety, menopause transition, and other psychological symptoms;^57,62,63^ and decreasing anxiety symptoms with age.^64,65^ Fortunately, quality evidence exists for anxiety treatment in older females. Pharmacological management of anxiety can include SSRIs, SNRIs, and benzodiazepine anxiolytics.^66,67^ Menopausal hormone therapy may be helpful when anxiety coincides with frequent VMS.^67^ Psychotherapies, including CBT, discussion groups, and relaxation training,^68^ can also be successful in older females,^69^ particularly because depressive symptoms and loneliness are strong predictors of generalized anxiety disorder symptom severity.^70^ Lavender oil (silexan),^66,71^ chamomile oil,^71^ and physical activity^72^ are additional anxiety treatments for older females.

### Influence of education

Education level most influences menopause therapy effectiveness beliefs and symptom treatment choices. Higher education attainment increased the likelihood of choosing exercise or mind–body therapies to treat menopause symptoms, participants with college degrees were more likely to choose exercise, and those with graduate degrees were more likely to choose mind–body therapies. Similar findings indicate higher CIT use in affluent, educated, white, postmenopausal females with nonprivate insurance and excellent or very good self-reported health.^13,73^ Perhaps higher-educated females are more aware of menopausal symptoms and treatment strategies, and thus more likely to seek symptom treatment,^74^ or have greater access to information and financial resources to use CIT.

### Physician recommendations

Participants primarily relied on doctors' recommendations and research to aid in CIT use decision-making, which is well supported in the literature.^13^ Unfortunately, poor or biased communication from health care providers can result in patients feeling ill-informed about CIT options,^13^ potentially resulting in use of less reliable information sources and failure to disclose CIT use.

Racial, ethnic, and socioeconomic differences may also influence decision-making.^75–77^ African-, Hispanic-, and Asian American females experience, report, and treat menopause symptoms differently and may choose to rely on information from elders or close friends. In addition, they may choose more holistic treatments over medication management or may avoid seeking care and discussing CIT options with an ill-informed health care provider.^78^ It is imperative that health care providers stay current on evidence-based CIT and MHT recommendations, exercise use for symptom management, and engage patients in culturally sensitive conversations and education regarding CIT use for menopause symptom treatment.

### Strengths and limitations

There are several strengths of this study. This study included a large sample size. Participants had a wide variety of options for mind–body therapies (*n* = 19), diet (*n* = 14), integrative therapies (*n* = 13), and exercise (*n* = 11) in a well-validated survey.^15^ Clear descriptions allowed the survey to capture more clearly how participants used different CIT. Furthermore, while participants knew that researchers were from the University of Minnesota, minimal information regarding researcher backgrounds was shared during data collection, reducing potential bias toward specific treatments.

There were also several limitations. As with any self-reported survey, information may be incomplete, over-reported, or under-reported. There was also a risk that participants were using CIT for symptoms that may be directly related but not attributed to menopause. Furthermore, the large number of CIT assessed limited more specific data collection, and information was not collected on past versus current treatments, use of multiple CIT simultaneously, and if use of particular CIT was linked with specific symptoms.

An additional and important limitation of this study was the lack of sample population diversity. Use of a convenience sample from the Minnesota State Fair resulted in a population that was predominantly white, highly educated and compensated, employed, married, Christian, and primarily from either Minnesota or Wisconsin (85%). The absence of ethnic and racial minorities in this study may have led to over- or under-estimation of CIT use, and any regional differences in CIT use could not be observed. Ethnic, racial, and regional differences in CIT use^79–81^ have proven to be critically important considerations when treating female patients. Larger, national multicenter studies would improve understanding of CIT use in the United States.

## Conclusion

This study supports previous findings that the majority of peri- and post-menopausal females are using at least one type of CIT to treat their menopause-related symptoms. In addition, this study suggests that exercise and mind–body therapies were most utilized to treat sleep disturbances, depression, and anxiety. Furthermore, education levels influenced perception of CIT effectiveness and choices; doctor recommendations and research studies/evidence were valuable in aiding participant decision-making process.

Despite reported reliance on quality resources when choosing CIT for menopause symptom treatment, education regarding evidence-based supported therapies for symptom treatment, such as SSRI for depression and anxiety, or CBT-I for sleep difficulties, is necessary to ensure that females make well-informed decisions. Additional research needs to be conducted with more geographically, financially, ethnically, and racially diverse populations. In the interim, comprehensive, personalized care that considers the best available evidence-based therapies will provide the best care for all female patients.

## Abbreviations Used

CBT: cognitive behavioral therapy
CBT-I: CBT for insomnia
CIT: complementary and integrative therapies
MDD: major depressive disorder
MENQOL: menopause quality of life
MHT: menopausal hormone therapy
OR: odds ratio
SNRIs: serotonin and norepinephrine reuptake inhibitors
SSRIs: selective serotonin reuptake inhibitors
VMS: vasomotor symptoms

## References

[1] Marlatt KL, Beyl RA, Redman LM. A qualitative assessment of health behaviors and experiences during menopause: A cross-sectional, observational study. Maturitas 2018;116:36–42. 30244777 10.1016/j.maturitas.2018.07.014 PMC6223619

[2] Monteleone P, Mascagni G, Giannini A, et al. Symptoms of menopause—Global prevalence, physiology and implications. Nat Rev Endocrinol 2018;14(4):199–215. 29393299 10.1038/nrendo.2017.180

[3] North American Menopause Society. The 2017 Hormone Therapy Position Statement of the North American Menopause Society. Menopause 2018;25(11):1362–1387. 30358733 10.1097/GME.0000000000001241

[4] Bruce D, Rymer J. Symptoms of the menopause. Best Pract Res Clin Obstet Gynaecol 2009;23(1):25–32. 19056320 10.1016/j.bpobgyn.2008.10.002

[5] Rossouw JE, Anderson GL, Prentice RL, et al. Risks and benefits of estrogen plus progestin in healthy postmenopausal women: Principal results from the women's health initiative randomized controlled trial. JAMA 2002;288(3):321–333. 12117397 10.1001/jama.288.3.321

[6] Langer RD, Hodis HN, Lobo RA, et al. Hormone replacement therapy—Where are we now? Climacteric 2021;24(1):3–10. 33403881 10.1080/13697137.2020.1851183

[7] Lambrinoudaki I. Menopausal hormone therapy and breast cancer risk: All progestogens are not the same. Case Rep Womens Health 2021;29:e00270. 33294389 10.1016/j.crwh.2020.e00270 PMC7683312

[8] Mehta JM, Chester RC, Kling JM. The timing hypothesis: Hormone therapy for treating symptomatic women during menopause and its relationship to cardiovascular disease. J Women's Health (Larchmt) 2019;28(5):705–711. 30484736 10.1089/jwh.2018.7201

[9] Anderson GL, Limacher M, Assaf AR, et al. Effects of conjugated equine estrogen in postmenopausal women with hysterectomy: The women's health initiative randomized controlled trial. JAMA 2004;291(14):1701–1712. 15082697 10.1001/jama.291.14.1701

[10] Nelson HD, Humphrey LL, Nygren P, et al. Postmenopausal hormone replacement therapy: Scientific review. JAMA 2002;288(7):872–881. 12186605 10.1001/jama.288.7.872

[11] Gentry-Maharaj A, Karpinskyj C, Glazer C, et al. Prevalence and predictors of complementary and alternative medicine/non-pharmacological interventions use for menopausal symptoms within the UK Collaborative Trial of ovarian cancer screening. Climacteric 2017;20(3):240–247. 28326899 10.1080/13697137.2017.1301919 PMC5448394

[12] Posadzki P, Lee MS, Moon TW, et al. Prevalence of complementary and alternative medicine (CAM) use by menopausal women: A systematic review of surveys. Maturitas 2013;75(1):34–43. 23497959 10.1016/j.maturitas.2013.02.005

[13] Peng W, Adams J, Sibbritt DW, et al. Critical review of complementary and alternative medicine use in menopause: Focus on prevalence, motivation, decision-making, and communication. Menopause 2014;21(5):536–548. 24104604 10.1097/GME.0b013e3182a46a3e

[14] Anonymous. Menopause Glossary. n.d. Available from: https://www.menopause.org/for-women/menopause-glossary [Last accessed: July 20, 2022].

[15] Tracy MF, Lindquist R, Savik K, et al. Use of complementary and alternative therapies: A national survey of critical care nurses. Am J Crit Care 2005;14(5):404–414. 16120892

[16] Berman BM, Singh BB, Hartnoll SM, et al. Primary care physicians and complementary-alternative medicine: Training, attitudes, and practice patterns. J Am Board Fam Pract 1998;11(4):272–281. 9719349 10.3122/jabfm.11.4.272

[17] National Center for Complementary and Integrative Health. Complementary, Alternative, or Integrative Health: What's in a Name? n.d. Available from: https://www.nccih.nih.gov/health/complementary-alternative-or-integrative-health-whats-in-a-name [Last accessed: October 26, 2022].

[18] Radtke JV, Terhorst L, Cohen SM. The menopause-specific quality of life questionnaire: Psychometric evaluation among breast cancer survivors. Menopause 2011;18(3):289–295. 20881889 10.1097/gme.0b013e3181ef975a PMC3017657

[19] Hilditch JR, Lewis J, Peter A, et al. A menopause-specific quality of life questionnaire: Development and psychometric properties. Maturitas 1996;24(3):161–175. 8844630 10.1016/s0378-5122(96)82006-8

[20] Asikainen T-M, Kukkonen-Harjula K, Miilunpalo S. Exercise for health for early postmenopausal women. Sports Med 2004;34(11):753–778. 15456348 10.2165/00007256-200434110-00004

[21] Sydora BC, Turner C, Malley A, et al. Can walking exercise programs improve health for women in menopause transition and postmenopausal? Findings from a scoping review. Menopause 2020;27(8):952–963. 32404793 10.1097/GME.0000000000001554

[22] Flesaker MQ, Serviente C, Troy LM, et al. The role of cardiorespiratory fitness on quality of life in midlife women. Menopause 2021;28(4):431–438. 33438890 10.1097/GME.0000000000001719 PMC9585341

[23] Stojanovska L, Apostolopoulos V, Polman R, et al. To exercise, or, not to exercise, during menopause and beyond. Maturitas 2014;77(4):318–323. 24548848 10.1016/j.maturitas.2014.01.006

[24] Oliveira D, Hachul H, Tufik S, et al. Effect of massage in postmenopausal women with insomnia: A pilot study. Clinics 2011;66(2):343–346. 21484056 10.1590/S1807-59322011000200026 PMC3059875

[25] Hachul H, Oliveira DS, Bittencourt LRA, et al. The beneficial effects of massage therapy for insomnia in postmenopausal women. Sleep Sci 2014;7(2):114–116. 26483913 10.1016/j.slsci.2014.09.005 PMC4521661

[26] Garcia MC, Kozasa EH, Tufik S, et al. The effects of mindfulness and relaxation training for insomnia (MRTI) on postmenopausal women: A pilot study. Menopause 2018;25(9):992–1003. 29787483 10.1097/GME.0000000000001118

[27] Jorge MP, Santaella DF, Pontes IMO, et al. Hatha yoga practice decreases menopause symptoms and improves quality of life: A randomized controlled trial. Complement Ther Med 2016;26:128–135. 27261993 10.1016/j.ctim.2016.03.014

[28] Stefanopoulou E, Grunfeld EA. Mind-body interventions for vasomotor symptoms in healthy menopausal women and breast cancer survivors. A systematic review. J Psychosom Obstet Gynaecol 2017;38(3):210–225. 27832718 10.1080/0167482X.2016.1235147

[29] van Driel CM, Stuursma A, Schroevers MJ, et al. Mindfulness, cognitive behavioural and behaviour-based therapy for natural and treatment-induced menopausal symptoms: A systematic review and meta-analysis. BJOG 2019;126(3):330–339. 29542222 10.1111/1471-0528.15153 PMC6585818

[30] Kravitz HM, Ganz PA, Bromberger J, et al. Sleep difficulty in women at midlife: A community survey of sleep and the menopausal transition. Menopause 2003;10(1):19–28. 12544673 10.1097/00042192-200310010-00005

[31] Kravitz HM, Joffe H. Sleep during the perimenopause: A SWAN Story. Obstet Gynecol Clin North Am 2011;38(3):567–586. 21961720 10.1016/j.ogc.2011.06.002 PMC3185248

[32] Smith RL, Flaws JA, Mahoney MM. Factors associated with poor sleep during menopause: Results from the midlife women's health study. Sleep Med 2018;45:98–105. 29680438 10.1016/j.sleep.2018.01.012 PMC5918428

[33] de Zambotti M, Sizintsev M, Claudatos S, et al. Reducing bedtime physiological arousal levels using immersive audio-visual respiratory bio-feedback: A pilot study in women with insomnia symptoms. J Behav Med 2019;42(5):973–983. 30790211 10.1007/s10865-019-00020-9 PMC6703979

[34] Bromberger JT, Kravitz HM, Youk A, et al. Patterns of depressive disorders across 13 years and their determinants among midlife women: SWAN Mental Health Study. J Affect Disord 2016;206:31–40. 27455356 10.1016/j.jad.2016.07.005 PMC5077630

[35] Thurston RC, Chang Y, von Känel R, et al. Sleep characteristics and carotid atherosclerosis among midlife women. Sleep 2017;40(2); doi: 10.1093/sleep/zsw052. PMC6084762 28364498

[36] Matthews KA, Everson-Rose SA, Kravitz HM, et al. Do reports of sleep disturbance relate to coronary and aortic calcification in healthy middle-aged women?: Study of women's health across the nation. Sleep Med 2013;14(3):282–287. 23352420 10.1016/j.sleep.2012.11.016 PMC3582843

[37] Jehan S, Masters-Isarilov A, Salifu I, et al. Sleep disorders in postmenopausal women. J Sleep Disord Ther 2015;4(5):212. 26512337 PMC4621258

[38] Vincent AJ, Ranasinha S, Sayakhot P, et al. Sleep difficulty mediates effects of vasomotor symptoms on mood in younger breast cancer survivors. Climacteric 2014;17(5):598–604. 24673553 10.3109/13697137.2014.900745

[39] Baker FC, de Zambotti M, Colrain IM, et al. Sleep problems during the menopausal transition: Prevalence, impact, and management challenges. Nat Sci Sleep 2018;10:73–95. 29445307 10.2147/NSS.S125807 PMC5810528

[40] Hachul H, Polesel DN. Insomnia pharmacotherapy: A review of current treatment options for insomnia in menopause. Curr Sleep Med Rep 2017;3(4):299–305.

[41] Berin E, Hammar M, Lindblom H, et al. Effects of resistance training on quality of life in postmenopausal women with vasomotor symptoms. Climacteric 2022;25(3):264–270; doi: 10.1080/13697137.2021.1941849. 34240669

[42] Rubio-Arias JÁ, Marín-Cascales E, Ramos-Campo DJ, et al. Effect of exercise on sleep quality and insomnia in middle-aged women: A systematic review and meta-analysis of randomized controlled trials. Maturitas 2017;100:49–56. 28539176 10.1016/j.maturitas.2017.04.003

[43] Soares CN. Depression and menopause: An update on current knowledge and clinical management for this critical window. Med Clin North Am 2019;103(4):651–667. 31078198 10.1016/j.mcna.2019.03.001

[44] Soares CN, Shea AK. The midlife transition, depression, and its clinical management. Obstet Gynecol Clin North Am 2021;48(1):215–229. 33573787 10.1016/j.ogc.2020.11.009

[45] Freeman EW, Sammel MD, Lin H, et al. Associations of hormones and menopausal status with depressed mood in women with no history of depression. Arch Gen Psychiatry 2006;63(4):375–382. 16585466 10.1001/archpsyc.63.4.375

[46] Bromberger JT, Schott L, Kravitz HM, et al. Risk factors for major depression during midlife among a community sample of women with and without prior major depression: Are they the same or different? Psychol Med 2015;45(8):1653–1664. 25417760 10.1017/S0033291714002773 PMC4414245

[47] Maki PM, Kornstein SG, Joffe H, et al. Guidelines for the evaluation and treatment of perimenopausal depression: Summary and recommendations. J Women's Health (Larchmt) 2019;28(2):117–134. 30182804 10.1089/jwh.2018.27099.mensocrec

[48] Hollon SD, DeRubeis RJ, Fawcett J, et al. Effect of cognitive therapy with antidepressant medications vs antidepressants alone on the rate of recovery in major depressive disorder: A randomized clinical trial. JAMA Psychiatry 2014;71(10):1157–1164. 25142196 10.1001/jamapsychiatry.2014.1054 PMC4315327

[49] Stute P, Spyropoulou A, Karageorgiou V, et al. Management of depressive symptoms in peri- and postmenopausal women: EMAS Position Statement. Maturitas 2020;131:91–101. 31740049 10.1016/j.maturitas.2019.11.002

[50] Soares CN, Zitek B. Reproductive hormone sensitivity and risk for depression across the female life cycle: A continuum of vulnerability? J Psychiatry Neurosci 2008;33(4):331–343. 18592034 PMC2440795

[51] Soares CN. Mood disorders in midlife women: Understanding the critical window and its clinical implications. Menopause 2014;21(2):198–206. 24448106 10.1097/GME.0000000000000193

[52] Hodis HN, Mack WJ. A “Window of Opportunity”: The reduction of coronary heart disease and total mortality with menopausal therapies is age- and time-dependent. Brain Res 2011;1379:244–252. 20977895 10.1016/j.brainres.2010.10.076 PMC3046231

[53] de Novaes Soares C, Almeida OP, Joffe H, et al. Efficacy of estradiol for the treatment of depressive disorders in perimenopausal women: A double-blind, randomized, placebo-controlled trial. Arch Gen Psychiatry 2001;58(6):529–534. 11386980 10.1001/archpsyc.58.6.529

[54] Gordon JL, Rubinow DR, Eisenlohr-Moul TA, et al. Efficacy of transdermal estradiol and micronized progesterone in the prevention of depressive symptoms in the menopause transition: A randomized clinical trial. JAMA Psychiatry 2018;75(2):149–157. 29322164 10.1001/jamapsychiatry.2017.3998 PMC5838629

[55] Morrison MF, Kallan MJ, Ten Have T, et al. Lack of efficacy of estradiol for depression in postmenopausal women: A randomized, controlled trial. Biol Psychiatry 2004;55(4):406–412. 14960294 10.1016/j.biopsych.2003.08.011

[56] Avis NE, Stellato R, Crawford S, et al. Is there a menopausal syndrome? Menopausal status and symptoms across racial/ethnic groups. Soc Sci Med 2001;52(3):345–356. 11330770 10.1016/s0277-9536(00)00147-7

[57] Bryant C, Judd FK, Hickey M. Anxiety during the menopausal transition: A systematic review. J Affect Disord 2012;139(2):141–148. 21783260 10.1016/j.jad.2011.06.055

[58] Sigmon ST, Dorhofer DM, Rohan KJ, et al. The impact of anxiety sensitivity, bodily expectations, and cultural beliefs on menstrual symptom reporting: A test of the menstrual reactivity hypothesis. J Anxiety Disord 2000;14(6):615–633. 11918095 10.1016/s0887-6185(00)00054-2

[59] Reynolds F. Relationships between catastrophic thoughts, perceived control and distress during menopausal hot flushes: Exploring the correlates of a questionnaire measure. Maturitas 2000;36(2):113–122. 11006498 10.1016/s0378-5122(00)00142-0

[60] Bromberger JT, Kravitz HM, Chang Y, et al. Does risk for anxiety increase during the menopausal transition? Study of women's health across the nation. Menopause 2013;20(5):488–495. 23615639 10.1097/GME.0b013e3182730599 PMC3641149

[61] Jeste DV, Blazer DG, First M. Aging-related diagnostic variations: Need for diagnostic criteria appropriate for elderly psychiatric patients. Biol Psychiatry 2005;58(4):265–271. 16102544 10.1016/j.biopsych.2005.02.004

[62] Hardy R, Kuh D. Change in psychological and vasomotor symptom reporting during the menopause. Soc Sci Med 2002;55(11):1975–1988. 12406465 10.1016/s0277-9536(01)00326-4

[63] Bryant C, Jackson H, Ames D. The prevalence of anxiety in older adults: Methodological issues and a review of the literature. J Affect Disord 2008;109(3):233–250. 18155775 10.1016/j.jad.2007.11.008

[64] Jorm AF. Does old age reduce the risk of anxiety and depression? A review of epidemiological studies across the adult life span. Psychol Med 2000;30(1):11–22. 10722172 10.1017/s0033291799001452

[65] Andreescu C, Lee S. Anxiety disorders in the elderly. In: Anxiety Disorders: Rethinking and Understanding Recent Discoveries. (Kim Y-K. ed.) Springer Singapore: Singapore; 2020; pp. 561–576. 10.1007/978-981-32-9705-0_28 32002946

[66] Strawn JR, Geracioti L, Rajdev N, et al. Pharmacotherapy for generalized anxiety disorder in adult and pediatric patients: An evidence-based treatment review. Expert Opin Pharmacother 2018;19(10):1057–1070. 30056792 10.1080/14656566.2018.1491966 PMC6340395

[67] Siegel AM, Mathews SB. Diagnosis and treatment of anxiety in the aging woman. Curr Psychiatry Rep 2015;17(12):93. 26458819 10.1007/s11920-015-0636-3

[68] Klainin-Yobas P, Oo WN, Suzanne Yew PY, et al. Effects of relaxation interventions on depression and anxiety among older adults: A systematic review. Aging Ment Health 2015;19(12):1043–1055. 25574576 10.1080/13607863.2014.997191

[69] Gonçalves DC, Byrne GJ. Interventions for generalized anxiety disorder in older adults: Systematic review and meta-analysis. J Anxiety Disord 2012;26(1):1–11. 21907538 10.1016/j.janxdis.2011.08.010

[70] Boehlen FH, Herzog W, Schellberg D, et al. Gender-specific predictors of generalized anxiety disorder symptoms in older adults: Results of a large population-based study. J Affect Disord 2020;262:174–181. 31668601 10.1016/j.jad.2019.10.025

[71] Ebrahimi H, Mardani A, Basirinezhad MH, et al. The effects of lavender and chamomile essential oil inhalation aromatherapy on depression, anxiety and stress in older community-dwelling people: A randomized controlled trial. Explore 2022;18(3):272–278. 33454232 10.1016/j.explore.2020.12.012

[72] Mochcovitch MD, Deslandes AC, Freire RC, et al. The effects of regular physical activity on anxiety symptoms in healthy older adults: A systematic review. Braz J Psychiatry 2016;38(3):255–261. 27579597 10.1590/1516-4446-2015-1893 PMC7194273

[73] Schonberg MA, Wee CC. Menopausal symptom management and prevention counseling after the women's health initiative among women seen in an internal medicine practice. J Women's Health (Larchmt) 2005;14(6):507–514. 16115005 10.1089/jwh.2005.14.507

[74] Namazi M, Sadeghi R, Behboodi Moghadam Z. Social determinants of health in menopause: An integrative review. Int J Women's Health 2019;11:637–647. 31849539 10.2147/IJWH.S228594 PMC6910086

[75] Helenius IM, Korenstein D, Halm EA. Changing use of hormone therapy among minority women since the women's health initiative. Menopause 2007;14(2):216–222. 17179789 10.1097/01.gme.0000233169.65045.b1

[76] Paramsothy P, Harlow SD, Nan B, et al. Duration of the menopausal transition is longer in women with young age at onset: The multiethnic study of women's health across the nation. Menopause 2017;24(2):142–149. 27676632 10.1097/GME.0000000000000736 PMC5266650

[77] Kuh DL, Wadsworth M, Hardy R. Women's health in midlife: The influence of the menopause, social factors and health in earlier life. Br J Obstet Gynaecol 1997;104(8):923–933. 9255084 10.1111/j.1471-0528.1997.tb14352.x

[78] Richard-Davis G, Wellons M. Racial and ethnic differences in the physiology and clinical symptoms of menopause. Semin Reprod Med 2013;31(5):380–386. 23934699 10.1055/s-0033-1348897

[79] Malika NM, Desai AK, Belliard JC. Herbal use and medical pluralism among Latinos in Southern California. J Community Health 2017;42(5):949–955. 28364317 10.1007/s10900-017-0340-9

[80] Rhee TG, Evans RL, McAlpine DD, et al. Racial/ethnic differences in the use of complementary and alternative medicine in US adults with moderate mental distress. J Prim Care Community Health 2017;8(2):43–54. 27678243 10.1177/2150131916671229 PMC5932659

[81] Zhang Y, Dennis JA, Leach MJ, et al. Complementary and alternative medicine use among US adults with headache or migraine: Results from the 2012 National Health Interview Survey. Headache 2017;57(8):1228–1242. 28742215 10.1111/head.13148

